# Case report: Immune pressure on hematopoietic stem cells can drastically expand glycosylphosphatidylinositol-deficient clones in paroxysmal nocturnal hemoglobinuria

**DOI:** 10.3389/fimmu.2023.1329403

**Published:** 2024-01-15

**Authors:** Naoki Shingai, Hiroki Mizumaki, Yuho Najima, Yuta Yamada, Dung Cao Tran, Kyoko Haraguchi, Takashi Toya, Yoshiki Okuyama, Noriko Doki, Yasuhito Nannya, Seishi Ogawa, Shinji Nakao

**Affiliations:** ^1^ Hematology Division, Tokyo Metropolitan Cancer and Infectious Diseases Center, Komagome Hospital, Tokyo, Japan; ^2^ Department of Hematology, Kanazawa University, Kanazawa, Japan; ^3^ Division of Transfusion and Cell Therapy, Tokyo Metropolitan Cancer and Infectious Diseases Center, Komagome Hospital, Tokyo, Japan; ^4^ Department of Pathology and Tumor Biology, Graduate School of Medicine, Kyoto University, Kyoto, Japan

**Keywords:** paroxysmal nocturnal hemoglobinuria (PNH), bone marrow failure (BMF), syngeneic hematopoietic stem cell transplantation, glycosylphosphatidylinositol (GPI) anchor, phosphatidylinositol N-acetylglucosaminyltransferase subunit A (PIGA)

## Abstract

**Introduction:**

Paroxysmal nocturnal hemoglobinuria (PNH) is a rare hematological disease characterized by intravascular hemolysis, thrombosis, and bone marrow (BM) failure. Although PNH is caused by excessive proliferation of hematopoietic stem cell (HSC) clones with loss of function mutations in phosphatidylinositol N-acetylglucosaminyltransferase subunit A (*PIGA*) genes, what drives PNH clones to expand remains elusive.

**Case description:**

We present a case of a 26-year-old female who presented with hemolytic anemia, thrombocytopenia, and leukopenia. Flow cytometry analysis of peripheral blood showed that 71.9% and 15.3% of the granulocytes and erythrocytes were glycosylphosphatidylinositol-anchored protein deficient (GPI[-]) cells. The patient was diagnosed with PNH with non-severe aplastic anemia. Deep-targeted sequencing covering 390 different genes of sorted GPI(-) granulocytes revealed three different *PIGA* mutations (p.I69fs, variant allele frequency (VAF) 24.2%; p.T192P, VAF 5.8%; p.V300fs, VAF 5.1%) and no other mutations. She received six cycles of eculizumab and oral cyclosporine. Although the patient’s serum lactate dehydrogenase level decreased, she remained dependent on red blood cell transfusion. Six months after diagnosis, she received a syngeneic bone marrow transplant (BMT) from a genetically identical healthy twin, following an immune ablative conditioning regimen consisting of cyclophosphamide 200 mg/kg and rabbit anti-thymocyte globulin 10 mg/kg. After four years, the patient’s blood count remained normal without any signs of hemolysis. However, the peripheral blood still contained 0.2% GPI (-) granulocytes, and the three *PIGA* mutations that had been detected before BMT persisted at similar proportions to those before transplantation (p.I69fs, VAF 36.1%; p.T192P, VAF 3.7%; p.V300fs, VAF 8.6%) in the small PNH clones that persisted after transplantation.

**Conclusions:**

The PNH clones that had increased excessively before BMT decreased, but persisted at low percentages for more than four years after the immunoablative conditioning regimen followed by syngeneic BMT. These findings indicate that as opposed to conventional theory, immune pressure on HSCs, which caused BM failure before BMT, was sufficient for *PIGA*-mutated HSCs to clonally expand to develop PNH.

## Introduction

Paroxysmal nocturnal hemoglobinuria (PNH) is a rare hematological disease characterized by intravascular hemolysis, thrombosis, and bone marrow (BM) failure. Phosphatidylinositol N-acetylglucosaminyltransferase subunit A (*PIGA*) is the only gene on the X chromosome involved in glycosylphosphatidylinositol (GPI) biosynthesis. Somatic loss of function mutations in *PIGA* results in the loss of CD55 and CD59, key complemental regulators on the surface of red blood cells, leading to intravascular hemolysis (1). The clinical manifestations of PNH occur when hematopoietic stem cells (HSCs) carrying somatic *PIGA* mutations expand excessively (2). The HSCs carrying these mutations are thought to be initially selected immunologically before they acquire driver mutations in some genes, such as *HMGA2*, *JAK2*, and *BCR-ABL*, which provide these HSCs with a proliferative advantage over normal HSCs (3–5). However, the mechanisms underlying excessive proliferation of *PIGA*-mutated HSCs are largely unknown as such driver gene mutations have been detected only in a limited number of cases of PNH, and previous studies using whole exome sequencing have failed to detect somatic mutations in sorted GPI-anchored protein deficient (GPI[-]) granulocytes in most patients with PNH (4). Immune pressure on HSCs may be sufficient for *PIGA*-mutated HSCs to clonally expand, causing PNH, but this remains unproven.

Herein, we present a case where original GPI-deficient PNH clones persisted after syngeneic BM transplantation (BMT) but did not expand within four and a half years.

## Case description

**Figure 1:**
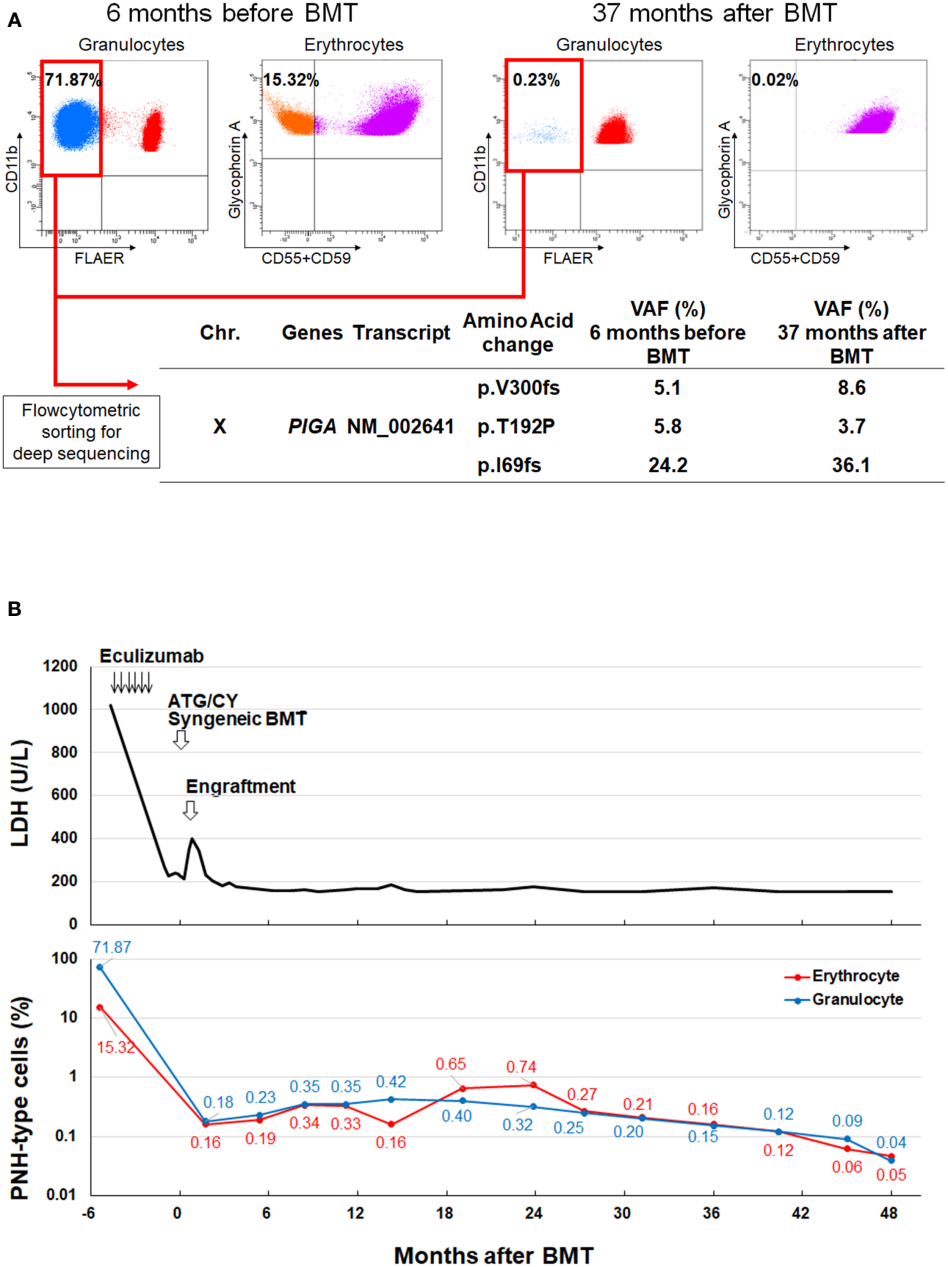
PNH clones before and after syngeneic BMT. **(A)** GPI (–) granulocytes and erythrocytes 6 months before (Upper left) and 37 months after BMT (Upper right). Deep-targeted sequencing of GPI (–) granulocytes sorted from the same blood samples revealed three different identical *PIGA* mutations (Lower). **(B)** Changes in the LDH level and the percentage of GPI (–) cells before and after syngeneic BMT. BMT, bone marrow transplantation; Chr, chromosome; GPI, glycosylphosphatidylinositol; LDH, lactate dehydrogenase, PNH, paroxysmal nocturnal hemoglobinuria; VAF, variant allele frequency.

**Figure 2:**
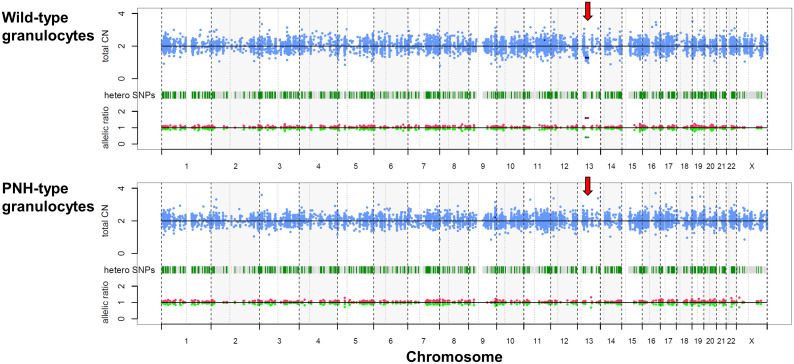
Copy number alteration of sorted granulocytes. Copy number alteration analysis of flowcytometric-sorted granulocytes from peripheral blood six months before bone marrow transplantation. Red arrows indicate the deletion of chromosome 13q in the wild-type granulocytes (upper), while it is not evident in the GPI (–) granulocytes (lower). CN, copy number; PNH, paroxysmal nocturnal hemoglobinuria; SNP, single nucleotide polymorphism.

A 26-year-old female presented with hemolytic anemia, thrombocytopenia, and leukopenia (hemoglobin level 5.8 g/dL, platelet count 26 × 10^9^/L, white blood cell count 2.3 × 10^9^/L, absolute neutrophil count 1.1 × 10^9^/L; **Table 1**). Flow cytometry analysis of peripheral blood showed that 71.9% and 15.3% of the granulocytes and erythrocytes were GPI (–) (**Figure 1A**). The bone marrow biopsy at diagnosis showed decreased myelopoiesis and megakaryopoiesis without significant evidence of dysplasia. G-banding of BM cells revealed the presence of del (13q) in five of the twenty metaphases. The patient was diagnosed with PNH with non-severe aplastic anemia (AA). Deep-targeted sequencing covering 390 different genes of GPI (–) granulocytes revealed three different *PIGA* mutations (p.I69fs, variant allele frequency (VAF) 24.2%; p.T192P, VAF 5.8%; p.V300fs, VAF 5.1%; **Figure 1A**) and no other mutations (**Table 2**). Copy number alteration analysis of flow cytometric-sorted granulocytes revealed del(13q) in wild-type granulocytes but not in GPI (–) granulocytes (**Figure 2**). Details of materials and methods are provided in the **Supplementary Methods**. The treatment included six cycles of eculizumab and oral cyclosporine. Although the patient’s serum lactate dehydrogenase level decreased to 267 units/L and her reticulocyte count increased to 110 × 10^9^/L after treatment, she remained dependent on red blood cell transfusions, probably due to extravascular hemolysis. Her platelet count and absolute neutrophil count were still low at 29 × 10^9^/L and 0.9 × 10^9^/L regardless of cyclosporine therapy. Hematopoietic stem cell transplantation from her monozygotic twin was therefore considered necessary to achieve hematologic remission. It was found that the patient’s healthy twin did not harbor GPI (–) cells. Six months after diagnosis, syngeneic BMT was performed following an immune ablative conditioning regimen (cyclophosphamide (CY) 200 mg/kg and rabbit anti-thymocyte globulin (ATG) 10 mg/kg). Calcineurin inhibitors were not used. Neutrophil and platelet engraftment occurred on days 12 and 20, respectively, and the patient’s blood cell count normalized on day 341. However, flow cytometry performed on day 341 revealed 0.35% granulocytes and 0.33% erythrocytes to be GPI (–). At that time, we expected that the PNH clones would expand sooner or later, leading to relapse of PNH because they were thought to have an intrinsic growth advantage, given the high percentage of PNH-type granulocytes before syngeneic BMT. However, the percentages of GPI (–) granulocytes and erythrocytes remained almost the same thereafter (**Figure 1B**). At four and a half years, the patient’s blood cell counts were normal, without any signs of hemolysis. Chromosomal analysis of BM showed a normal karyotype. The percentages of GPI (–) granulocytes and erythrocytes were 0.04% and 0.05%, respectively (**Figure 1B**). Deep sequencing of the sorted GPI (–) granulocytes using amplified *PIGA* exons revealed the same three *PIGA* mutations at proportions similar to those before transplantation (p.I69fs, VAF 36.1%; p.T192P, VAF 3.7%; p.V300fs, VAF 8.6%; **Figure 1A**) in the small PNH clones that persisted after transplantation. Although donor/recipient chimerism could not be measured due to the syngeneic nature of the transplant, donor chimerism is estimated to be very high considering the disappearance of del(13q) and the very low fraction of residual PNH clones. The patient is happy with her current condition, as she was told by her physician that her PNH could eventually relapse because small PNH clones persisted after BMT.

**Table 1:** Laboratory data at diagnosis.

Complete blood count		Blood chemistry			
WBC	2.3×10^9^ /L	TP	7.0 g/dL	Blood glucose	107 mg/dL
Blast	0 %	ALB	4.5 g/dL	HbA1c	4.0 %
Myelo	0 %	BUN	12.7 mg/dL	T chol	131 mg/dL
Stab	0 %	Cr	0.54 mg/dL	LDL chol	48 mg/dL
Seg	47 %	UA	3.8 mg/dL	TG	44 mg/dL
Lymph	47 %	Na	138 mEq/L	Free T3	2.94 pg/mL
Mono	4 %	K	4.0 mEq/L	Free T4	1.2 ng/mL
Eos	2 %	Cl	109 mEq/L	TSH	2.28 μIU
Baso	0 %	Ca	8.8 mg/dL	BNP	31.9 pg/mL
Hb	5.8 g/dL	T-bil	1.03 mg/dL	Fe	149 μg/dL
RBC	1.50×10^12^ /L	D-bil	0.05 mg/dL	UIBC	227 μg/dL
Ht	17.4 g/dL	I-bil	0.98 mg/dL	Ferritin	17.6 ng/mL
MCV	116 fL	AST	35 U/L	**Coagulation study**	
MCH	38.7 pg	ALT	10 U/L	PT	107 %
MCHC	33.3 %	LDH	841 U/L	PT-INR	0.95
Reticulocytes	4.7 %	ALP	222 U/L	APTT	22.8 s
PLT	26×10^9^ /L	γ-GTP	14 U/L	Fib	309 mg/dL
IPF	2.0 %	CRP	0.03 mg/dL	FDP	2.4 μg/mL

**Table 2:** Deep-targeted sequencing of flow cytometric-sorted GPI(-) granulycytes.

Time Point	Chromosome	Start	End	Ref.	Alt.	Type	Genes	Transcript	Amino Acid change	VAF (%)
6 month before BMT	chrX	15343225	15343225	-	T	Frameshift insertion	PIGA	NM_002641	p.V300fs	5.1
	chrX	15349479	15349479	T	G	Nonsynonymous SNV	PIGA	NM_002641	p.T192P	5.8
	chrX	15349846	15349846	A	-	Frameshift deletion	PIGA	NM_002641	p.I69fs	24.2
37 months after BMT	chrX	15343225	15343225	-	T	Frameshift insertion	PIGA	NM_002641	p.V300fs	8.6
	chrX	15349479	15349479	T	G	Nonsynonymous SNV	PIGA	NM_002641	p.T192P	3.7
	chrX	15349846	15349846	A	-	Frameshift deletion	PIGA	NM_002641	p.I69fs	36.1

BMT, bone marrow transplantation; chr, chromosome; GPI, glycosylphosphatidylinositol; SNV, single nucleotide variant; PIGA, phosphatidylinositol N-acetylglucosaminyltransferase subunit A; VAF, variant allele frequency.

## Discussion and conclusions

In this case, the GPI-deficient PNH clones that had increased excessively before BMT markedly decreased after the immunoablative conditioning regimen followed by syngeneic BMT but did not disappear. Cho et al. (6) reported a patient with PNH who underwent syngeneic peripheral blood stem cell transplantation following conditioning with CY 200 mg/kg alone. The patient showed remission of PNH, but eventually relapsed 12 months after transplant. It was expected that the PNH clones of our patient would gradually expand to cause a relapse of PNH because they had the same *PIGA* mutations as the PNH clones that showed excessive proliferation before BMT. However, this was not the case. The addition of rabbit ATG to CY may have abolished immune mechanisms that confer a survival advantage on *PIGA*-mutated HSCs.

One might assume that an intensified conditioning regimen is required to eliminate the large PNH clone in patients with PNH who undergo syngeneic BMT. Our experience in this case suggests that elimination of the immune mechanism would be sufficient to induce durable remission of PNH. Oved et al. (7) reported a case of secondary PNH that occurred 10 years after allo-BMT from an HLA-matched sibling donor for treatment of PNH with AA. Although the patient’s PNH clone was only 0.2% of the granulocyte population when he developed donor-type aplasia 5 years after allo-BMT, the recipient-derived PNH granulocytes increased to 4.49% 14 years after BMT, which was associated with symptomatic hemolysis. Because our patient received the same conditioning regimen as the case reported by Oved et al, her PNH clones may grow again if she relapsed with AA. In addition, new PNH clone, different from the original PNH clones that existed prior to syngeneic BMT, may emerge when PNH relapses (8). Therefore, we need to carefully monitor the patients for the occurrence of pancytopenia, which may indicate a relapse of PNH.

GPI-deficient HSCs have no intrinsic growth advantage and do not expand clonally over time. A previous study (9) using CRISPR/Cas9 gene editing suggested that *PIGA* mutations in long-term viable HSCs are insufficient to induce rapid clonal expansion or a PNH disease phenotype in a macaque model. Very few granulocytes with *PIGA* mutations are commonly detected even in healthy individuals (10); however, GPI-deficient clones do not expand sufficiently to cause PNH symptoms (11).

Several immunological proof-of-concept scenarios have been reported in which GPI-deficient HSCs may expand (12), including natural killer (NK) cell-mediated cytotoxicity (13) NK T cells targeting GPI bound to CD1d molecules (14), and CD4^+^ T cell-mediated immunologic attack (15). A mouse model study showed that GPI (–) cells continued to proliferate in the presence of CD4^+^ T cells and remained non-proliferative in their absence (15). Immunosuppressive therapy can cause a reduction in PNH clone size in patients with AA (16). Indeed, one study reported that PNH clones became undetectable in 10 out of 83 patients after successful immunosuppressive therapy (16). It has also been reported that the proportion of PNH clones decreases when replaced by clonal hematopoietic stem cells of indeterminate potential or secondary myeloid neoplasm clones with other genetic mutations (17). In our case, next generation sequencing did not reveal any genetic mutations other than *PIGA* mutations in cells of peripheral blood obtained before syngeneic BMT, and no secondary myeloid neoplasm was observed during long-term follow-up. Despite the persistence of the same *PIGA*-mutated HSCs as those detected before BMT, the small PNH clones remained inactive for more than 4 years. These findings suggest that immune pressure on HSCs is sufficient for *PIGA*-mutated HSCs to clonally expand to cause PNH in at least some cases.

The limitation of this study is that we were unable to provide direct evidence to support that the immunoablative regimen eliminated the immune mechanism that allowed PNH HSCs to proliferate excessively because pre-transplant blood samples were not stored. Investigation of case similar to ours is warranted to uncover the immune mechanisms that give PNH HSCs a proliferative advantage.

## Data availability statement

The original contributions presented in the study are included in the article/**Supplementary Material**, further inquiries can be directed to the corresponding author/s.

## Ethics statement

The studies involving humans were approved by Institutional Review Board and Ethics Committee of Kanazawa University. The studies were conducted in accordance with the local legislation and institutional requirements. The participants provided their written informed consent to participate in this study. Written informed consent was obtained from the individual(s) for the publication of any potentially identifiable images or data included in this article.

## Author contributions

NS: Writing – original draft, Writing – review & editing, Data curation, Formal analysis, Investigation, Visualization. HM: Data curation, Formal analysis, Investigation, Methodology, Visualization, Writing – original draft, Writing – review & editing. YuN: Conceptualization, Data curation, Funding acquisition, Investigation, Project administration, Supervision, Visualization, Writing – original draft, Writing – review & editing. YY: Data curation, Investigation, Methodology, Writing – review & editing. DT: Formal analysis, Methodology, Visualization, Writing – review & editing. KH: Data curation, Formal analysis, Investigation, Methodology, Writing – review & editing. TT: Data curation, Investigation, Writing – review & editing. YO: Data curation, Formal analysis, Investigation, Methodology, Writing – review & editing. ND: Data curation, Investigation, Writing – review & editing. YaN: Data curation, Formal analysis, Methodology, Visualization, Writing – review & editing. SO: Data curation, Formal analysis, Methodology, Writing – review & editing. SN: Conceptualization, Data curation, Formal analysis, Funding acquisition, Investigation, Methodology, Project administration, Supervision, Writing – review & editing, Writing – original draft.
