# Supplementary material for: Case report: Immune pressure on hematopoietic stem cells can drastically expand glycosylphosphatidylinositol-deficient clones in paroxysmal nocturnal hemoglobinuria
Source: Front Immunol. 2024 Jan 15;14:1329403. doi: 10.3389/fimmu.2023.1329403 (PMC10822943; doi:10.3389/fimmu.2023.1329403)
Supplement: Supplementary file 1 [file DataSheet_1.pdf]

Supplemental information

Immune pressure on hematopoietic stem cells can drastically expand glycosylphosphatidylinositol-deficient clones in paroxysmal nocturnal hemoglobinuria

SUPPLEMENTAL METHODS

Flow cytometry assay

High-sensitivity flow cytometry assay was performed as previously described[1]. Briefly, we position PNH-type cells in the upper left quadrant (ULQ) of the scatterplot after gating mature granulocytes/erythrocytes using high expression of lineage specific markers. Then, we determine the positivity of PNH type cells using a qualitative line, followed by determining the exact percentage of PNH type cells, which includes Type III and Type II PNH cells, using a quantitative line.

Sample preparation for cell sorting

Leukocytes from the patient were treated with PE-labeled anti-CD55 monoclonal antibodies (mAbs) and anti-CD59 mAbs, and CD55+CD59+ granulocytes were removed using magnetic microbeads labeled with anti-PE mAbs (Anti-PE MicroBeads, Miltenyi Biotec, Gaithersburg, MD, USA). Using a fluorescence-activated cell sorter (BD FACSAria™ Fusion, BD Biosciences, Franklin Lakes, NJ, USA), paired fractions of CD11b+FLAER-negative granulocytes (GPI[-] Gs) and CD11b+FLAER-positive granulocytes (GPI[+] Gs) were sorted from granulocytes. NucleoSpin® Tissue XS (Takara bio, Shiga, Japan) was used to extract genomic DNA from GPI(-) Gs and GPI(+) Gs. The DNA samples were subjected to next-generation sequencing (NGS).

Deep-targeted sequencing

The genomic DNA from GPI(-) Gs and GPI(+) Gs were subjected to targeted capture sequencing as previously described[2, 3]. RNA baits were designed for detection of oncogenic variants in 390 known driver genes implicated myeloid malignancies. A custom complementary RNA bait library to capture

these 390 genes was generated using SureSelect custom kit (Agilent Technologies, Santa Clara, CA, USA). Additional baits for 1158 single-nucleotide polymorphisms (SNPs) were also included to calculated genome-wide copy numbers. These probes were deliberately selected so that they cover the human genome uniformly to allow for prospective detection of copy-number change and loss of heterozygosity (LOH) on the NGS platform. Copy-number abnormalities were identified using the data of allele frequencies and sequenced depth of SNPs.

PIGA Amplicon Sequencing

With a next generation sequencer (MiSeq; illumina, San Diego, CA, USA), nucleotide sequences of *PIGA* in sorted granulocytes of the patient were determined by deep sequencing of long-range PCR amplicons of *PIGA* as previously described[1]. Briefly, PCR mixtures containing genomic DNA, 0.2 μM primers in 20 μL of 1 × PrimeSTAR GXL DNA Polymerase (Takara Bio) were used to amplify each exon of the *PIGA* gene individually. Cycling conditions were as follows; 35 cycles of 98°C for 10 sec, 60°C for 15 sec, and 68°C for 3 min. Each *PIGA* exon amplicon was mixed and subjected to library construction for *PIGA* amplicon sequencing. The MiSeq sequencer was used to obtain paired-end sequence reads (150 bp read 1 and 151 bp read 2 in length). Somatic mutations were detected as difference from reference sequence with allele frequency (AF <2%), and covering with at least 1000 reads. Paired-end illumine reads were mapped to the reference genome (GRCh37) using Burrows-Wheeler Aligner (bwa) v.0.7.17[4]. SAM files generated by bwa were converted to the BAM format, then sorted and indexed using SAM tools v.1.9[5]. Duplicated reads were marked with Picard v.2.26 (https://github.com/broadinstitute/picard). The heuristic somatic mutation caller, VarScan 2, was used to detect somatic mutation after alignment of reads[6]. Alignment data from granulocytes were visually compared using the Integrative Genomics Viewer (IGV) and mutations were verified using the Unified Genotyper in the Genome Analysis Toolkit (GATK) v4.18[7, 8]. Somatic mutations were given functional annotation and COSMIC (https://cancer.sanger.ac.uk/cosmic) mutation ID meaning by ANNOVAR[9].

References

1. Hosokawa K, Sugimori C, Ishiyama K, Takamatsu H, Noji H, Shichishima T, *et al*. Establishment of a flow cytometry assay for detecting paroxysmal nocturnal hemoglobinuria-type cells specific to patients with bone marrow failure. *Ann Hematol* 2018 Dec; **97**(12) :2289-2297.

2. Yoshizato T, Nannya Y, Atsuta Y, Shiozawa Y, Iijima-Yamashita Y, Yoshida K, *et al*. Genetic abnormalities in myelodysplasia and secondary acute myeloid leukemia: impact on outcome of stem cell transplantation. *Blood* 2017 Apr 27; **129**(17): 2347-2358.

3. Mizumaki H, Tran DC, Hosokawa K, Hosomichi K, Zaimoku Y, Takamatsu H, *et al*. Minor GPI(-) granulocyte populations in aplastic anemia and healthy individuals derived from a few PIGA-mutated hematopoietic stem progenitor cells. *Blood Cancer J* 2023 Nov 8; **13**(1): 165.

4. Li H, Durbin R. Fast and accurate short read alignment with Burrows-Wheeler transform. *Bioinformatics* 2009 Jul 15; **25**(14): 1754-1760.

5. Li H, Handsaker B, Wysoker A, Fennell T, Ruan J, Homer N, *et al*. The Sequence Alignment/Map format and SAMtools. *Bioinformatics* 2009 Aug 15; **25**(16): 2078-2079.

6. Koboldt DC, Zhang Q, Larson DE, Shen D, McLellan MD, Lin L, *et al*. VarScan 2: somatic mutation and copy number alteration discovery in cancer by exome sequencing. *Genome Res* 2012 Mar; **22**(3): 568-576.

7. McKenna A, Hanna M, Banks E, Sivachenko A, Cibulskis K, Kernytsky A, *et al*. The Genome Analysis Toolkit: a MapReduce framework for analyzing next-generation DNA sequencing data. *Genome Res* 2010 Sep; **20**(9): 1297-1303.

8. Robinson JT, Thorvaldsdottir H, Winckler W, Guttman M, Lander ES, Getz G, *et al*. Integrative genomics viewer. *Nat Biotechnol* 2011 Jan; **29**(1): 24-26.

9. Wang K, Li M, Hakonarson H. ANNOVAR: functional annotation of genetic variants from high-throughput sequencing data. *Nucleic Acids Res* 2010 Sep; **38**(16): e164.
